# Clinical and genetic profile of patients enrolled in the Transthyretin Amyloidosis Outcomes Survey (THAOS): 14-year update

**DOI:** 10.1186/s13023-022-02359-w

**Published:** 2022-06-18

**Authors:** Angela Dispenzieri, Teresa Coelho, Isabel Conceição, Márcia Waddington-Cruz, Jonas Wixner, Arnt V. Kristen, Claudio Rapezzi, Violaine Planté-Bordeneuve, Juan Gonzalez-Moreno, Mathew S. Maurer, Martha Grogan, Doug Chapman, Leslie Amass, the THAOS investigators, Pablo Garcia Pavia, Ivaylo Tarnev, Jose Gonzalez Costello, Maria Alejandra Gonzalez Duarte Briseno, Hartmut Schmidt, Brian Drachman, Fabio Adrian Barroso, Taro Yamashita, Olivier Lairez, Yoshiki Sekijima, Giuseppe Vita, Eun-Seok Jeon, Mazen Hanna, David Slosky, Marco Luigetti, Samantha LoRusso, Francisco Munoz Beamud, David Adams, Henning Moelgaard, Rayomand Press, Calogero Lino Cirami, Hans Nienhuis, Josep Maria Campistol Plana, Jocelyn Inamo, Daniel Jacoby, Michele Emdin, Dianna Quan, Scott Hummel, Ronald Witteles, Amir Dori, Sanjiv Shah, Daniel Lenihan, Olga Azevedo, Srinivas Murali, Sasa Zivkovic, Soon Chai Low, Jose Nativi-Nicolau, Nowell Fine, Jose Tallaj, Carsten Tschoepe, Roberto Fernandéz Torrón, Michael Polydefkis, Giampaolo Merlini, Sorina Badelita, Stephen Gottlieb, James Tauras, Edileide Barros Correia, Hector Ventura, Burkhard Gess, Felix Darstein, Jeeyoung Oh, Tessa Marburger, Johan Van Cleemput, Valeria Lujan Salutto, Yesim Parman, Chi-Chao Chao, Nitasha Sarswat, Christopher Mueller, David Steidley, Jeffrey Ralph, Alberta Warner, William Cotts, James Hoffman, Marcelo Rugiero, Sonoko Misawa, Jose Luis Munoz Blanco, Lucia Galan Davila, Menachem Sadeh, Jin Luo, Theodoros Kyriakides, Annabel Wang, Horacio Kaufmann, Sasa Zivkovic

**Affiliations:** 1 Division of Hematology, Mayo Clinic, Rochester, MN USA; 2 Unidade Corino Andrade, Hospital Santo António, Centro Hospitalar Universitário do Porto, Porto, Portugal; 3 Department of Neurosciences, CHULN, Hospital de Santa Maria, Universidade de Lisboa, Lisbon, Portugal; 4 University Hospital, Federal University of Rio de Janeiro, National Amyloidosis Referral Center, CEPARM, Rio de Janeiro, Brazil; 5 Department of Public Health and Clinical Medicine, Umeå University, Umeå, Sweden; 6 Department of Cardiology, Angiology, Respiratory Medicine, Medical University of Heidelberg, Heidelberg, Germany; 7 Cardiological Centre, University of Ferrara, Ferrara, Italy; 8 Maria Cecilia Hospital, GVM Care & Research, Cotignola, Ravenna, Italy; 9 Hôpital Henri Mondor – AP-HP, East Paris University, Créteil, France; 10 Servicio de Medicina Interna, Hospital Universitario Son Llatzer, Instituto de Investigación Sanitaria Illes Balears, Palma de Mallorca, Spain; 11 Columbia University College of Physicians and Surgeons, New York, NY USA; 12 Department of Cardiovascular Diseases, Mayo Clinic, Rochester, MN USA; 13 Pfizer Inc, New York, NY USA; 14 Hospital Universitario Puerta de Hierro, Majadahonda, Spain; 15 Alexandrovska University Hospital Clinic of Neurology, Sofia, Bulgaria; 16 Hospital Universitari de Bellvitge, Barcelona, Spain; 17 Instituto Nacional de Ciencia Medicas y Nutricion Salvador Zubiran, Distrito Federal, Mexico; 18 Universitatsklinikum Muenster – Transplant Hepatology, Muenster, Germany; 19 University of Pennsylvania – Perelman Center for Advanced Medicine, Philadelphia, PA USA; 20 FLENI, Ciudad Autonoma de Buenos Aires, Argentina; 21 Kumamoto University, Kumamoto-City, Japan; 22 CHU de Toulouse – Hôpital Rangueil, Toulouse, France; 23 Shinshu University School of Medicine, Matsumoto, Japan; 24 AOU Policlinico G. Martino – Messina – Dr. Vita, Messina, Italy; 25 Samsung Medical Center, Sungkyunkwan University School of Medicine, Seoul, Republic of Korea; 26 Cleveland Clinic Foundation, Cleveland, OH USA; 27 Vanderbilt University School of Medicine, Nashville, TN USA; 28 Fondazione Policlinico Gemelli, Universita Cattolica del Sacro Cuore, Rome, Italy; 29 The Ohio University College of Medicine, Columbus, OH USA; 30 Hospital Juan Ramon Jimenez, Huelva, Spain; 31 CHU de Bicêtre, Paris, France; 32 Aarhus University Hospital, Skejby, Aarhus, Denmark; 33 Karolinska University Hospital, Stockholm, Sweden; 34 Azienda Ospedaliero-Universitaria Di Careggi, Florence, Italy; 35 University Medical Center Groningen, Groningen, The Netherlands; 36 Institut Clinic de Nefrologia i Urologia – ICNU, Hospital Clinic i Provincial de Barcelona, Barcelona, Spain; 37 CHU de Fort-de-France, Fort-de-France, France; 38 Smilow Cancer Hospital at Yale-New Haven, New Haven, CT USA; 39 Fondazione Toscana Gabriele Monasterio Per La Ricerca Medica E Di Sanita Pubblica, Pisa, Italy; 40 UC Denver, Aurora, CO USA; 41 University of Michigan, Ann Arbor, MI USA; 42 Stanford University School of Medicine, Stanford, CA USA; 43 Sheba Medical Center, Ramat Gan, Israel; 44 Northwestern University, Chicago, IL USA; 45 Washington University School of Medicine, St. Louis, WA USA; 46 Centro Hospitalar Do Alto Ave, Epe, Guimaraes, Portugal; 47 Wexford Health and Wellness Pavillion, Pittsburgh, PA USA; 48 University of Pittsburgh Medical Center (UPMC), Pittsburgh, PA USA; 49 University Malaya Medical Centre (UMMC), Kuala Lumpur, Malaysia; 50 The University of Utah Health Sciences Center, Salt Lake City, UT USA; 51 University of Alberta Foothills Medical Centre, Calgary, AB Canada; 52 University of Alabama at Birmingham, Birmingham, AL USA; 53 Charité Campus Rudolf-Virchow-Klinikum, Berlin, Germany; 54 Hospital Universitario Donostia, San Sebastian, Gipuzkoa Spain; 55 Johns Hopkins Hospital, Baltimore, MD USA; 56 Centro per lo Studio e la Cura delle Amiloidosi Sistemiche, Pavia, Italy; 57 Institutul de Cardiologie Prof. Dr. C. C. Iliescu Spitalului Fundeni, Bucharest, Romania; 58 University of Maryland, Baltimore, MD USA; 59 Montefiore Medical Center-Jack D. Weiler Hospital, Bronx, NY USA; 60 Instituto Dante Pazzanese De Cardiologia, Sao Paulo, Brazil; 61 John Ochsner Heart & Vascular Institute, New Orleans, LA USA; 62 University Hospital of RWTH Aachen, Aachen, Germany; 63 Johann-Gutenberg-Universität, Mainz, Germany; 64 Konkuk University Medical Center, Seoul, Republic of Korea; 65 Oregon Health and Science University, Portland, OR USA; 66 Afdeling Klinische Cardiologie, O&N I, Louvain, Belgium; 67 Instituto De Investigaciones Medicas Dr Alfredo Lanari, Buenos Aires, Argentina; 68 Department of Neurology, Istanbul University, Istanbul, Turkey; 69 National Taiwan University Hospital, Taipei, Taiwan; 70 University of Chicago Medical Center, Chicago, IL USA; 71 Medical College of Wisconsin, Milwaukee, WI USA; 72 Mayo Clinic Arizona, Phoenix, AZ USA; 73 Department of Neurology, University of CA – San Francisco, San Francisco, CA USA; 74 VA Greater Los Angeles Healthcare System, Los Angeles, CA USA; 75 Advocate Christ Medical Centre, Oak Lawn, IL USA; 76 University of Miami Hospital & Clinics, Miami, FL USA; 77 Hospital Italiano de Buenos Aires (HIBA), Buenos Aires, Argentina; 78 Chiba University Hospital, Chiba-shi, Japan; 79 Hospital Gregorio Marañón, Madrid, Spain; 80 Hospital Clinico San Carlos, Madrid, Spain; 81 Wolfson Medical Center, Holon, Israel; 82 Temple University School of Medicine, Philadelphia, PA USA; 83 Cyprus Institute of Neurology and Genetics, Nicosia, Cyprus; 84 University of California Irvine, Orange, CA USA; 85 NYU Medical Center, New York, NY USA; 86 University of Pittsburgh Medical Center (UPMC), Pittsburgh, USA

**Keywords:** Amyloidosis, Cardiomyopathy, Polyneuropathy, Transthyretin, Registry

## Abstract

**Background:**

Transthyretin amyloidosis (ATTR amyloidosis) is a rare, life-threatening disease caused by the accumulation of variant or wild-type (ATTRwt amyloidosis) transthyretin amyloid fibrils in the heart, peripheral nerves, and other tissues and organs.

**Methods:**

Established in 2007, the Transthyretin Amyloidosis Outcomes Survey (THAOS) is the largest ongoing, global, longitudinal observational study of patients with ATTR amyloidosis, including both inherited and wild-type disease, and asymptomatic carriers of pathogenic *TTR* mutations. This descriptive analysis examines baseline characteristics of symptomatic patients and asymptomatic gene carriers enrolled in THAOS since its inception in 2007 (data cutoff: August 1, 2021).

**Results:**

This analysis included 3779 symptomatic patients and 1830 asymptomatic gene carriers. Symptomatic patients were predominantly male (71.4%) and had a mean (standard deviation [SD]) age of symptom onset of 56.3 (17.8) years. Val30Met was the most common genotype in symptomatic patients in South America (80.9%), Europe (55.4%), and Asia (50.5%), and more patients had early- versus late-onset disease in these regions. The majority of symptomatic patients in North America (58.8%) had ATTRwt amyloidosis. The overall distribution of phenotypes in symptomatic patients was predominantly cardiac (40.7%), predominantly neurologic (40.1%), mixed (16.6%), and no phenotype (2.5%). In asymptomatic gene carriers, mean (SD) age at enrollment was 42.4 (15.7) years, 42.4% were male, and 73.2% carried the Val30Met mutation.

**Conclusions:**

This 14-year global overview of THAOS in over 5000 patients represents the largest analysis of ATTR amyloidosis to date and highlights the genotypic and phenotypic heterogeneity of the disease.

*ClinicalTrials.gov Identifier*: NCT00628745.

**Supplementary Information:**

The online version contains supplementary material available at 10.1186/s13023-022-02359-w.

## Introduction

Transthyretin amyloidosis (ATTR amyloidosis) is a multisystemic, life-threatening disease resulting from the deposition of transthyretin (TTR) amyloid fibrils in the heart, peripheral nerves, and other tissues, leading mainly to polyneuropathy and/or cardiomyopathy [1]. There are two distinct forms of ATTR amyloidosis: hereditary or variant ATTR amyloidosis (ATTRv amyloidosis), which is caused by pathogenic mutations that destabilize the TTR protein, and wild-type ATTR amyloidosis (ATTRwt amyloidosis), which results from the accumulation of wild-type TTR protein [2, 3]. The phenotypic presentation of ATTRv amyloidosis is variable and can be predominantly neurologic, predominantly cardiac, or a mix of both neurologic and cardiac manifestations, depending on the particular *TTR* variant and other factors [2, 4]. So far, over 140 *TTR* variants have been identified [5]. ATTRwt amyloidosis most often presents as cardiomyopathy [3].

ATTR amyloidosis is a progressive disease with a poor prognosis when left untreated. Life expectancy ranges between 2 and 10 years after symptom onset and depends upon disease type and other factors [2, 3, 6]. Obtaining an accurate diagnosis of ATTR amyloidosis can be difficult due to the heterogeneity of the disease, low disease awareness, and clinical features that overlap with more common disorders [2, 6, 7]. Improved understanding of the disease can facilitate earlier identification and intervention with approved disease-modifying therapies.

Established in 2007, the Transthyretin Amyloidosis Outcomes Survey (THAOS) is the largest ongoing, global, longitudinal observational study of patients with ATTR amyloidosis, including both inherited and wild-type disease, and asymptomatic carriers of pathogenic *TTR* mutations (NCT00628745) [8]. By studying a large, global patient population, THAOS has provided valuable insights into ATTR amyloidosis and has highlighted the genotypic, phenotypic, and geographic heterogeneity of the disease [9–13]. The objective of this descriptive analysis was to examine baseline characteristics of symptomatic patients with ATTR amyloidosis and asymptomatic gene carriers enrolled in THAOS since its inception 14 years ago.

## Methods

### Study design and patient population

The study design and eligibility criteria of THAOS have been described [8]. All study sites received ethical or institutional review board approval prior to patient enrollment, and each patient provided written informed consent. The study followed the Good Pharmacoepidemiology Practice guidelines and the principles of the Declaration of Helsinki.

The analysis population consisted of all patients enrolled in THAOS (data cutoff date: August 1, 2021). Symptomatic patients were those with at least one symptom reported as definitely related to ATTR amyloidosis at enrollment. Asymptomatic gene carriers were those with a pathogenic disease-causing *TTR* genetic variant and no definitely ATTR amyloidosis–related symptom at enrollment, with the requirement that all symptoms be assessed at enrollment. Patients with no definitely ATTR amyloidosis–related symptoms at enrollment who were not assessed for all symptoms were considered to have a missing symptomatic status.

Demographics, clinical characteristics, and patient-reported outcomes collected at enrollment were analyzed in the overall cohort of symptomatic patients and by the following genotype subgroups: ATTRwt amyloidosis; Val30Met with early-onset disease (age ≤ 50 years, based on age at diagnosis); Val30Met with late-onset disease (age > 50 years); ‘cardiac mutations’ (Val122Ile [p.Val142Ile] [14], Leu111Met [p.Leu131Met] [15], Thr60Ala [p.Thr80Ala] [16], and Ile68Leu [p.Ile88Leu] [17]); and non-Val30Met excluding cardiac mutations.

Phenotype at enrollment was further analyzed in symptomatic patients by region (North America, South America, Europe, and Asia). Phenotype categories were defined as follows: Predominantly cardiac included patients with at least one of the following symptoms: heart failure, dyspnea, or abnormal electrocardiogram due to rhythm disturbance, and no more than mild neurologic or gastrointestinal (GI) symptoms (excluding erectile dysfunction, constipation, and carpal tunnel syndrome); cardiac symptoms did not need to be ongoing at a given visit. Predominantly neurologic included patients with neurologic or GI symptoms of any severity without heart failure, dyspnea, or abnormal electrocardiogram due to rhythm disturbance; neurologic and GI symptoms had to be ongoing. Mixed included patients with heart failure, dyspnea, or abnormal electrocardiogram due to rhythm disturbance and neurologic or GI symptoms of any severity but did not satisfy the criteria for predominantly cardiac or predominantly neurologic. No phenotype included all other symptomatic patients who did not meet criteria for any of these phenotypes. All patients with ATTRwt amyloidosis were classified as predominantly cardiac unless they had any neurologic symptoms definitely related to ATTR amyloidosis, in which case they were classified as mixed phenotype.

Symptoms at baseline were categorized as autonomic neuropathy, cardiac disorder, gastrointestinal manifestations, motor neuropathy, sensory neuropathy, and other. Autonomic neuropathy included dizziness, palpitations, dry eye, constipation, diarrhea, diarrhea/constipation, early satiety, fecal incontinence, nausea, vomiting, recurrent urinary tract infections, urinary incontinence, urinary retention, dyshidrosis, and erectile dysfunction; cardiac disorder included coronary artery disease, dyspnea, heart failure, myocardial infarction, rhythm disturbance, syncope, arterial hypertension, cardiomyopathy, and other cardiovascular disease. Gastrointestinal manifestations included constipation, diarrhea, diarrhea/constipation, early satiety, fecal incontinence, nausea, unintentional weight loss, and vomiting. Motor neuropathy included muscle weakness and walking disability. Sensory neuropathy included balance abnormality, neuropathic arthropathy, neuropathic pain/paresthesia, numbness, temperature or pain insensitivity, and tingling. Symptom categories were not mutually exclusive.

Demographics collected at enrollment were also analyzed in the overall cohort of asymptomatic gene carriers and by genotype category (Val30Met, cardiac mutations, and non-Val30Met excluding cardiac).

### Assessments

Patients’ ability to perform normal daily life activities and their need for assistance was assessed in symptomatic patients using the Karnofsky Performance Status Scale score, ranging from 10 (moribund; fatal processes progressing rapidly) to 100 (normal; no complaints). Neurologic impairment was measured in symptomatic patients using the derived Neuropathy Impairment Score in the Lower Limbs (NIS-LL; ranges from 0 to 88) [18]. Higher scores indicate greater impairment, and the NIS-LL scale includes reflex, motor, and sensory subscales. Modified Polyneuropathy Disability (mPND) scores were analyzed in symptomatic patients with a predominantly neurologic or mixed phenotype. The mPND score is a measure of walking disability and ranges from 0 to IV, where 0 indicates no sensory disturbances in the feet and able to walk without difficulty; I indicates sensory disturbance in the feet but preserved walking capacity; II indicates some difficulties walking, but can walk without aid; IIIa indicates 1 stick or crutch required for walking; IIIb indicates 2 sticks or crutches required for walking; and IV indicates patients confined to a wheelchair or bed.

Measures of cardiac disease in symptomatic patients were left ventricular (LV) septal thickness and LV ejection fraction. Additional cardiac findings, including troponin and N-terminal pro-B-type natriuretic peptide (NT-proBNP) levels and New York Heart Association (NYHA) functional class, were analyzed in symptomatic patients with a predominantly cardiac or mixed phenotype.

Quality of life (QoL) was assessed in symptomatic patients using the EQ-5D-3L and the Norfolk Quality of Life – Diabetic Neuropathy questionnaire. The EQ-5D-3L is a measure of self-reported health status. The first part assesses health on five dimensions (mobility, self-care, usual activities, pain/discomfort, and anxiety/depression), each with three levels: no problems, some problems, extreme problems/unable to. Health state profiles are assigned a summary index score ranging from 0 (death) to 1 (perfect health). The second part is a visual analog scale on which participants rate perceived health from 0 (worst) to 100 (best). The 35-item Norfolk Quality of Life – Diabetic Neuropathy questionnaire assesses diabetic neuropathy across five domains: physical functioning/large fiber neuropathy, activities of daily living, symptoms, small-fiber neuropathy, and autonomic neuropathy. Scores range from − 4 to 136, with higher scores indicating worse QoL.

### Statistical analyses

This was a descriptive analysis. Continuous data are presented as mean (standard deviation [SD]) or median (10th, 90th percentile), and categorical data are presented as count (percentage).

## Results

### Demographics and genotype

**Fig. 1:**
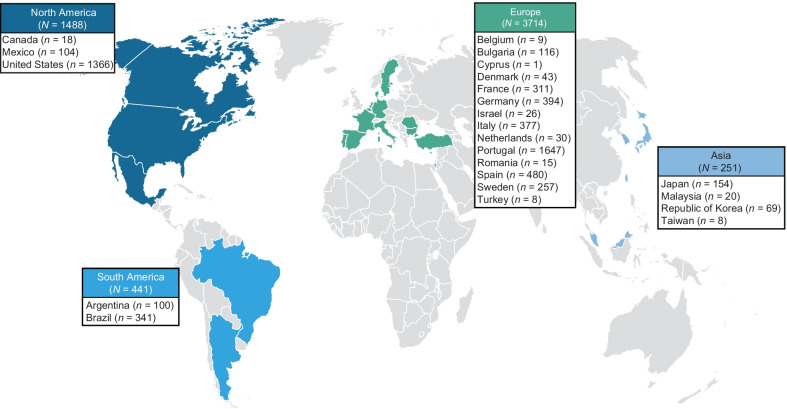
Geographic distribution of all patients enrolled in the Transthyretin Amyloidosis Outcomes Survey (THAOS)

There were 5894 patients from 84 study sites in 23 countries enrolled in THAOS at the data cutoff date (Fig. 1). Of these, 3779 were symptomatic patients, 1830 were asymptomatic gene carriers, and 61 were missing symptomatic status. Val30Met was the most prevalent genotype among all THAOS patients (49.6%), followed by ATTRwt amyloidosis (23.5%) and Val122Ile (6.0%) (Additional file 1). Val30Met was most common in Europe (64.6%), South America (80.3%), and Japan (74.0%), and wild-type disease was most common in North America (54.8%). Within Europe, non-Val30Met or cardiac mutations were more common in some individual countries (Bulgaria, Denmark, Israel, Italy, the Netherlands, Romania, and Turkey), but Val30Met was the predominant genotype overall.

**Fig. 2:**
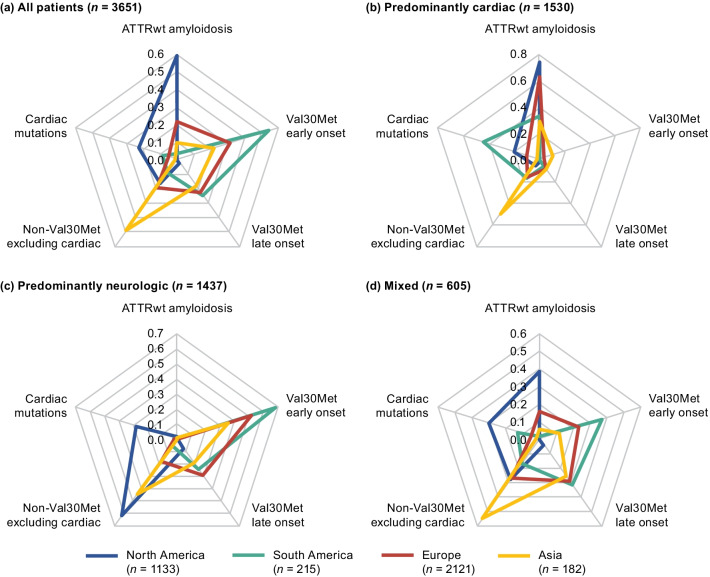
Regional distribution of genotype subgroups in symptomatic patients. The proportion of patients with each genotype shown by region in **a** the overall population of symptomatic patients and in patients with **b** predominantly cardiac, **c** predominantly neurologic, and **d** mixed phenotypes. The sum of values for each region in each figure equals one. Val30Met early onset and late onset *n* based on all patients with available data for disease diagnosis; 128 patients with the Val30Met mutation and no date of diagnosis were not included. Genotype categories of patients with no phenotype can be found in Additional file 2. Cardiac mutations include Val122Ile, Leu111Met, Thr60Ala, and Ile68Leu. ATTRwt amyloidosis = wild-type transthyretin amyloidosis

Symptomatic patients were predominantly male across all genotype subgroups (Table 1). Overall, the mean age at symptom onset was 56.3 years and was higher in patients with ATTRwt amyloidosis compared with the other genotype subgroups. Mean time from symptom onset to diagnosis was 4.0 years in all symptomatic patients and ranged from 2.0 years in the early-onset Val30Met subgroup to 4.7 years in the ATTRwt amyloidosis subgroup. The majority of patients in North America had ATTRwt amyloidosis (58.8%). Val30Met was the most common genotype in South America (80.9%), Europe (55.4%), and Asia (50.5%), and more patients had early- versus late-onset disease in each of these regions (Fig. 2a; Additional file 2).

**Table 1:** Demographics of symptomatic patients according to genotype category

	Overall (*n* = 3779)	ATTRwt amyloidosis (*n* = 1156)	Val30Met early onset (*n* = 826)	Val30Met late onset (*n* = 588)	Cardiac mutations (*n* = 384)	Non-Val30Met excluding cardiac (*n* = 697)
Male, n (%)	2698 (71.4)	1086 (93.9)	447 (54.1)	381 (64.8)	284 (74.0)	429 (61.5)
Race/ethnicity^a^, *n* (%)						
Caucasian	2083 (76.6)	961 (94.4)	183 (67.3)	336 (86.6)	157 (46.0)	397 (65.4)
African descent	269 (9.9)	30 (2.9)	26 (9.6)	16 (4.1)	164 (48.1)	28 (4.6)
American Hispanic	15 (0.6)	1 (0.1)	8 (2.9)	0	2 (0.6)	3 (0.5)
Latino American	124 (4.6)	5 (0.5)	18 (6.6)	4 (1.0)	15 (4.4)	78 (12.9)
Asian	218 (8.0)	15 (1.5)	37 (13.6)	31 (8.0)	2 (0.6)	99 (16.3)
Other	11 (0.4)	6 (0.6)	0	1 (0.3)	1 (0.3)	2 (0.3)
Age at enrollment (years), mean (SD)	62.3 (17.0)	77.5 (7.1)	39.8 (7.9)	67.9 (8.3)	69.3 (9.1)	56.9 (12.6)
Age at onset of ATTR amyloidosis symptoms (years), *n*	3775	1156	826	588	383	694
Mean (SD)	56.3 (17.8)	72.0 (9.7)	33.1 (6.5)	61.8 (9.3)	63.5 (11.3)	50.9 (12.6)
Time from symptom onset to diagnosis (years), *n*	3492	1092	826	588	347	639
Mean (SD)	4.0 (5.9)	4.7 (6.9)	2.0 (3.0)	4.4 (5.0)	4.6 (6.7)	4.6 (6.5)
Follow-up time^b^ (years), mean (SD)	3.6 (3.0)	2.0 (1.8)	6.2 (3.0)	3.9 (2.7)	2.4 (2.2)	3.1 (2.6)

Val30Met early onset and late onset *n* based on all patients with available data for disease diagnosis; 128 patients with Val30Met were missing date of diagnosis. Symptom onset was the date of first occurrence of symptom(s) reported as definitely related to ATTR amyloidosis. Cardiac mutations include Val122Ile, Leu111Met, Thr60Ala, and Ile68Leu

^a^Denominator for race/ethnicity is the total of non-missing records

^b^Follow-up time is based on all patients, from enrollment to last observation

ATTR amyloidosis = transthyretin amyloidosis; ATTRv amyloidosis = hereditary transthyretin amyloidosis; ATTRwt amyloidosis = wild-type transthyretin amyloidosis; SD = standard deviation

Of the 1830 asymptomatic gene carriers, 42.4% were male and 73.2% carried the Val30Met mutation (Table 2). Mean age at enrollment was 42.4 years and was higher in asymptomatic gene carriers with cardiac mutations than those in the other genotype subgroups.

**Table 2:** Demographics of asymptomatic gene carriers according to genotype category

	Overall (*n* = 1830)	Val30Met (*n* = 1339)	Cardiac mutations (*n* = 200)	Non-Val30Met excluding cardiac (*n* = 291)
Male, *n* (%)	776 (42.4)	514 (38.4)	115 (57.5%)	147 (50.5)
Race/ethnicity^a^, *n* (%)				
Caucasian	677 (77.9)	384 (87.3)	113 (60.1)	180 (74.7)
African descent	103 (11.9)	27 (6.1)	68 (36.2)	8 (3.3)
American Hispanic	11 (1.3)	7 (1.6)	2 (1.1)	2 (0.8)
Latino American	40 (4.6)	8 (1.8)	5 (2.7)	27 (11.2)
Asian	33 (3.8)	12 (2.7)	0	21 (8.7)
Other	5 (0.6)	2 (0.5)	0	3 (1.2)
Age at enrollment (years), mean (SD)	42.4 (15.7)	39.3 (14.5)	57.3 (16.1)	46.0 (14.1)

Cardiac mutations include Val122Ile, Leu111Met, Thr60Ala, and Ile68Leu

^a^Denominator for race/ethnicity is the total of non-missing records

SD = standard deviation

### Distribution of phenotypes at enrollment in symptomatic patients

**Fig. 3:**
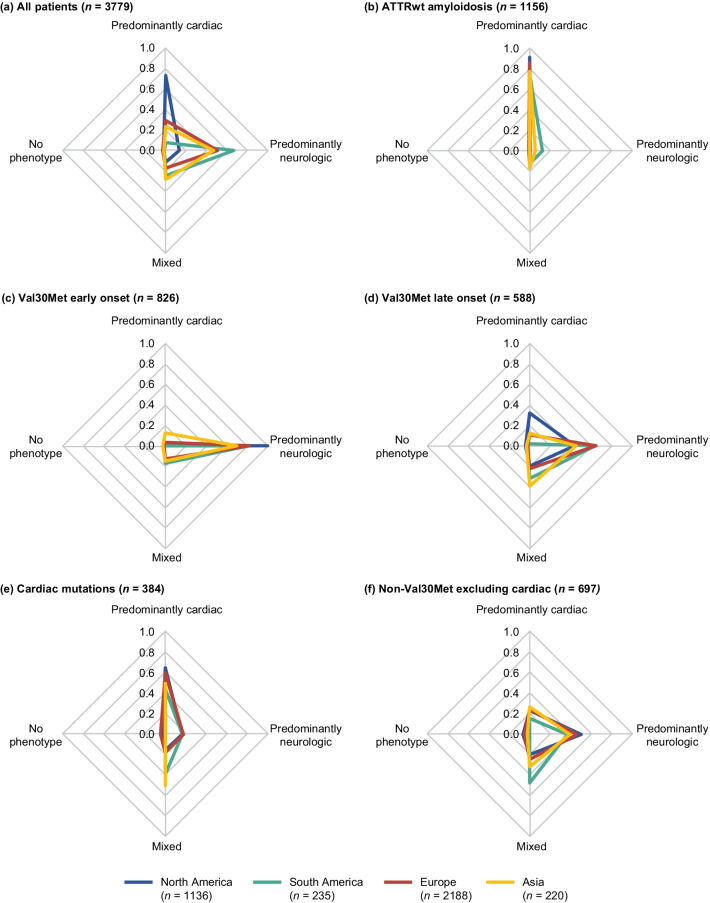
Regional distribution of phenotype at enrollment in symptomatic patients. The proportion of patients with each phenotype shown by region in **a** the overall population of symptomatic patients and by genotype category, **b** ATTRwt amyloidosis, **c** Val30Met early onset, **d** Val30Met late onset, **e** cardiac mutations, and **f** non-Val30Met excluding cardiac mutations. The sum of values for each region in each figure equals one. Val30Met early onset and late onset *n* based on all patients with available data for disease diagnosis; 128 patients with the Val30Met mutation and no date of disease diagnosis were not included in the genotype category breakdown. Cardiac mutations include Val122Ile, Leu111Met, Thr60Ala, and Ile68Leu. ATTRwt amyloidosis = wild-type transthyretin amyloidosis

The overall phenotype distribution for symptomatic patients at enrollment was predominantly cardiac (40.7%), predominantly neurologic (40.1%), mixed (16.6%), and no phenotype (2.5%) (Additional file 2). Predominantly cardiac was the most common phenotype at enrollment in North America, whereas predominantly neurologic was the most common phenotype at enrollment in Europe, South America, and Asia (Fig. 3a; Additional file 2).

ATTRwt amyloidosis comprised most of the predominantly cardiac phenotype patients in North America and Europe (Fig. 2b). For Asia and South America, respectively, the non-Val30Met excluding cardiac and cardiac mutations genotype subgroups were most common among the predominantly cardiac phenotype (Fig. 2b). The only region in which Val30Met (early or late onset) did not account for the majority of the predominantly neurologic phenotype was North America (Fig. 2c). More than half of patients with a mixed phenotype from Asia had non-Val30Met excluding cardiac as their genotype; 38.5% of the mixed phenotype in North America consisted of ATTRwt patients (Fig. 2d).

**Fig. 4:**
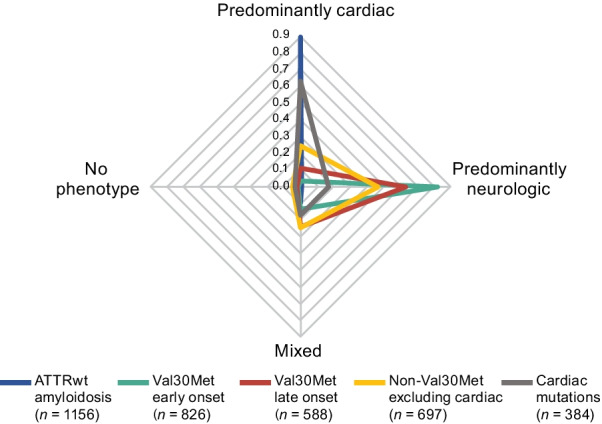
Distribution of phenotype at enrollment in symptomatic patients according to genotype category. The proportions of patients with each phenotype are shown by genotype. The sum of values for each genotype equals one. Val30Met early onset and late onset *n* based on all patients with available data for disease diagnosis; 128 patients with the Val30Met mutation and no date of diagnosis were not included. Cardiac mutations include Val122Ile, Leu111Met, Thr60Ala, and Ile68Leu. ATTRwt amyloidosis = wild-type transthyretin amyloidosis

Most symptomatic patients with Val30Met had a predominantly neurologic phenotype at enrollment (72.8%) (Additional file 2). The Val30Met late- versus early-onset group had a greater proportion of mixed (24.1% vs 13.6%) and predominantly cardiac (10.9% vs 3.3%) (Fig. 4; Additional file 2). Most symptomatic patients with ATTRwt amyloidosis had a predominantly cardiac phenotype (89.0%), with 9.8% of patients with ATTRwt amyloidosis presenting with a mixed phenotype (Fig. 4; Additional file 2). The distribution of phenotypes in early-onset Val30Met and wild-type patients was generally consistent across global regions (Fig. 3b, c; Additional file 2). Slightly higher rates of predominantly cardiac phenotypes were observed in the North American late-onset Val30Met group than other regions with the same late-onset Val30Met mutation (Fig. 3d). Patients with cardiac mutations had a predominantly neurologic phenotype in 16.7% and a mixed phenotype in 17.2% of patients (Fig. 4; Additional file 2). The large proportion of patients with cardiac mutations who had a predominantly neurologic or mixed phenotype was most notable in the Asian and South American groups (Fig. 3e), though sample sizes were small. Among the non-Val30Met excluding cardiac mutations genotype group, patients from South America were most likely to have a mixed phenotype and least likely to have a predominantly cardiac phenotype (Fig. 3f; Additional file 2).

### Clinical characteristics at enrollment in symptomatic patients

Greater neurologic impairment was observed in the Val30Met and non-Val30Met excluding cardiac subgroups (Additional file 3). Patients with late-onset Val30Met had the highest neurologic impairment as measured by the NIS-LL. Greater cardiac impairment was observed in patients with ATTRwt amyloidosis or cardiac mutations (Additional file 3).

**Fig. 5:**
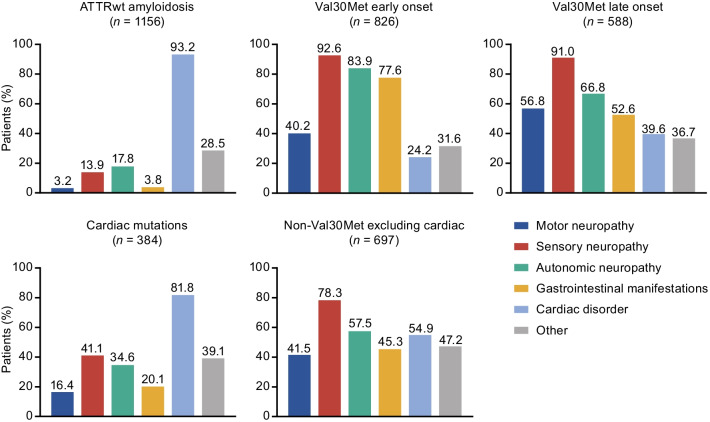
Symptom categories at enrollment in symptomatic patients according to genotype category.Val30Met early onset and late onset *n* based on all patients with available data for disease diagnosis; 128 patients with Val30Met and no date of disease diagnosis were not included. ATTRwt amyloidosis = wild-type transthyretin amyloidosis

Over half of symptomatic patients presented with sensory neuropathy (59.9%), cardiac disorder (59.4%), and/or autonomic neuropathy (50.1%) at enrollment. GI manifestations were present in 38.3% of patients, and motor neuropathy in 29.2% of patients. Cardiac disorder was the most common presenting symptom at enrollment in patients with ATTRwt amyloidosis or cardiac mutations, whereas sensory neuropathy was the most common presenting symptom in all other genotype subgroups (Fig. 5). Autonomic neuropathy and GI symptoms were more common in Val30Met early-onset as compared to late-onset patients, while motor neuropathy and cardiac disorder were more common in late-onset as compared to early-onset patients.

### Cardiac characteristics at enrollment in symptomatic patients with a predominantly cardiac or mixed phenotype

In the subset of patients with a predominantly cardiac or mixed phenotype, heart failure was present in 92.2% of patients with cardiac mutations, 88.6% of patients with ATTRwt amyloidosis, and 62.5% of patients with other mutations (Table 3). A greater proportion of patients with ATTRwt amyloidosis had an abnormal electrocardiogram, atrial fibrillation/flutter, and a pacemaker implanted than those with cardiac and other mutations.

**Table 3:** Cardiac characteristics at enrollment in symptomatic patients with a predominantly cardiac or mixed phenotype according to genotype category

	ATTRwt amyloidosis (*n* = 1142)	Cardiac mutations (*n* = 307)	Other mutations (*n* = 718)
Heart failure, *n* (%)	1012 (88.6)	283 (92.2)	449 (62.5)
NYHA functional class^a^, *n* (%)			
I	107 (10.5)	27 (9.5)	78 (17.4)
II	612 (59.8)	140 (49.3)	220 (49.1)
III	278 (27.2)	102 (35.9)	133 (29.7)
IV	26 (2.5)	15 (5.3)	17 (3.8)
Abnormal ECG, *n* (%)	947 (82.9)	218 (71.0)	415 (57.8)
Atrial fibrillation/flutter, *n* (%)	420 (36.8)	56 (18.2)	53 (7.4)
Pacemaker implanted, *n* (%)	179 (15.7)	26 (8.5)	33 (4.6)
ICD implanted, *n* (%)	59 (5.2)	16 (5.2)	12 (1.7)
NT-proBNP (pg/mL), *n*	775	127	253
Median (10th, 90th percentile)	2573.0 (731.0, 9041.0)	2648.0 (664.0, 8889.0)	1557.0 (213.0, 8633.0)
Troponin I (ng/mL), *n*	133	57	58
Median (10th, 90th percentile)	0.1 (0.0, 0.3)	0.1 (0.0, 0.5)	0.1 (0.0, 0.6)
Troponin T (ng/mL), *n*	626	86	215
Median (10th, 90th percentile)	0.1 (0.0, 0.2)	0.1 (0.0, 0.3)	0.0 (0.0, 0.1)

Cardiac mutations include Val122Ile, Leu111Met, Thr60Ala, and Ile68Leu. Other mutations include Val30Met and non-Val30Met excluding cardiac mutations

^a^Denominator for NYHA functional class is the total of non-missing records

ATTRwt amyloidosis = wild-type transthyretin amyloidosis; ECG = electrocardiogram; ICD = implantable cardioverter-defibrillator; NP-proBNP = N-terminal pro-B-type natriuretic peptide; NYHA = New York Heart Association; SD = standard deviation

### Neurologic characteristics at enrollment in symptomatic patients with a predominantly neurologic or mixed phenotype

Most patients with a predominantly neurologic or mixed phenotype had a score of I (52.1%) or II (20.7%) on the mPND (Additional file 4). Patients with Val30Met late-onset disease had greater walking impairment than those with Val30Met early-onset disease (mPND > II, 28.3% vs 8.0%).

## Discussion

This 14-year global overview of THAOS in over 5000 symptomatic patients with ATTR amyloidosis and asymptomatic gene carriers represents the largest descriptive analysis of the disease to date. Significant regional variation was observed in the distribution of genotypes and phenotypes. Val30Met was the most frequent variant genotype, and predominantly neurologic was the most frequent phenotype, in Europe, Asia, and South America, reflecting the endemic foci within these regions [2]. Alternatively, ATTRwt amyloidosis and cardiac mutations were the most common genotypes, and predominantly cardiac the most common phenotype, in North America, consistent with previously reported data [13].

Male predominance was observed in symptomatic patients across all genotypes, but most notably in patients with ATTRwt amyloidosis and cardiac mutations. These findings are consistent with the male predominance observed in ATTRwt amyloidosis, wherein men account for > 80% of diagnosed cases [22]. Mitochondrial DNA and neurohormonal factors may explain these sex-related differences [23, 24], although further studies are needed to clarify the role of these factors in the development of the disease. Although the proportion of males was greater than that of females in the early-onset Val30Met subgroup, the difference was not as great for this genotype subgroup (male, 54.1%; female, 45.9%). These results are in line with a prior report from a nationwide survey in Japan wherein the numbers of males and females with early-onset Val30Met disease were similar [19].

On the other hand, female predominance was observed among asymptomatic gene carriers. Prior reports suggest a later age of onset and lower disease penetrance in females, particularly in regard to cardiac manifestations [20, 21], and, therefore, females would be expected to represent a larger proportion of asymptomatic gene carriers.

Accurate diagnosis of ATTR amyloidosis can often be delayed for years. In this report, diagnosis of ATTR amyloidosis occurred an average of 4 years following symptom onset, with the longest time to diagnosis seen in patients with ATTRwt amyloidosis. Diagnosing ATTRwt amyloidosis can be particularly challenging because cardiac symptoms are often consistent with more common types of heart failure, and historically a diagnosis has been obtained through endomyocardial biopsy, an invasive and expensive technique [6, 25]. It is expected that increased use of bone scintigraphy, a minimally invasive and less expensive diagnostic tool, would decrease the time to diagnosis among these patients [3, 6], although this effect has not yet been reflected in THAOS [26]. Patients with early-onset Val30Met disease had the shortest time to diagnosis, but there was still an average 2 years from symptom onset to obtain a diagnosis despite most of these patients being from countries with endemic foci. This delay reflects the insidious onset of non-specific symptoms and underscores the need for objective biomarkers of onset and regular monitoring of asymptomatic carriers of pathogenic *TTR* mutations. Early identification of ATTR amyloidosis has become increasingly important with the recent approval of a number of disease-modifying therapies, including transthyretin stabilizers [27], small interfering RNA [28], and antisense oligonucleotides [29].

A mixed phenotype was observed in nearly 20% of symptomatic patients with Val30Met and cardiac mutations, and in ~ 10% of symptomatic patients with ATTRwt amyloidosis. Furthermore, patients presented with a variety of symptoms at enrollment. Autonomic neuropathy and/or sensory neuropathy were reported in ~ 15% of patients with ATTRwt amyloidosis and over one-third of patients with cardiac mutations, despite these mutations being associated primarily with cardiomyopathy. The majority of patients with Val30Met presented with sensory and autonomic neuropathy and GI manifestations. Motor neuropathy and cardiac disorder were also seen in a substantial proportion of Val30Met patients, with higher rates in those with late- versus early-onset disease. There has been increasing awareness of the multisystemic nature of ATTR amyloidosis, and the mixed phenotype may be more common than previously thought, including in patients with ATTRwt amyloidosis and with variants denoted as cardiac mutations in this analysis [12, 30–32]. Notably, 17% of patients with variants denoted as cardiac mutations had a predominantly neurologic phenotype. These findings are in line with recent reports of significant neurologic involvement in patients with mutations traditionally considered primarily cardiac [31].

The wide spectrum of symptoms observed at enrollment and the substantial proportion of patients with a mixed phenotype in this analysis emphasize the need for a multidisciplinary approach to the management of patients with all types of ATTR amyloidosis [33], and the importance of comprehensive evaluation, including neurologic, neurophysiological, and cardiac (electrocardiogram and echocardiogram) examinations. As evident in this study population, ATTR amyloidosis is a highly heterogeneous disease, and clinical manifestations can vary between different variants and/or geographic regions, and even between different family members who share the same pathogenic mutation.

### Strengths and limitations

A strength of the study was the inclusion of over 5000 patients from 23 countries, making this the largest descriptive analysis of ATTR amyloidosis to date. As with any disease registry, under-reporting of disease characteristics and under-ascertainment of patients are potential limitations that also apply to this THAOS analysis. Furthermore, the selection of patients included in THAOS may be biased, as there is an uneven distribution of specialties among investigators and study sites in some countries, and this could influence the phenotypic distribution across regions. For example, European patients are predominantly from Portugal, where two neurologic sites are the primary contributors of patients; and in the United States, cardiac sites are more common than neurologic sites. However, a prior analysis examined the relationship between the specialty of the investigator and the phenotype distribution of patients and found that, with a few exceptions, this relationship was not as strong as expected [12]. THAOS is a real-world registry so the proportion of neurologic, cardiologic, and other types of investigators will reflect the actual distribution of where patients with suspected ATTR amyloidosis are referred. There could also be under-reporting of mixed phenotypes in the earlier years of THAOS, before centers were consistently conducting comprehensive neurologic and cardiac assessments of all patients.

## Conclusion

This analysis of over 5000 symptomatic patients and asymptomatic *TTR* gene carriers demonstrates the wide heterogeneity and increasing awareness of ATTR amyloidosis. Although ATTR amyloidosis has historically been considered a primarily neurologic or a primarily cardiac disease, a mixed phenotype and multisystemic involvement are increasingly described. These findings highlight the need for a consistent, multidisciplinary approach to the management of ATTR amyloidosis.

## Supplementary Information

**Additional file 1: Table 1**. Most frequent genotypes recorded at enrollment in the overall population.

**Additional file 2: Table 2**. Distribution of phenotype in symptomatic patients according to genotype category.

**Additional file 3: Table 3**. Clinical characteristics and patient-reported outcomes in symptomatic patients according to genotype category.

**Additional file 4: Table 4**. Neurologic findings in symptomatic patients with a predominantly neurologic or mixed phenotype.

## Data Availability

Upon request, and subject to review, Pfizer will provide the data that support the findings of this study. Subject to certain criteria, conditions and exceptions, Pfizer may also provide access to the related individual de-identified participant data. See https://www.pfizer.com/science/clinical-trials/trial-data-and-results for more information.
