# Supplementary material for: Clinical and genetic profile of patients enrolled in the Transthyretin Amyloidosis Outcomes Survey (THAOS): 14-year update
Source: Orphanet J Rare Dis. 2022 Jun 18;17:236. doi: 10.1186/s13023-022-02359-w (PMC9206752; doi:10.1186/s13023-022-02359-w)
Supplement: Supplementary file 1 — Additional file 1: Table 1. Most frequent genotypes recorded at enrollment in the overall population. [file 13023_2022_2359_MOESM1_ESM.docx]

**Supplementary Table 1** Most frequent genotypes recorded at enrollment in the overall population

| **Genotype, *n* (%)** | **Overall**  **(*N* = 5894)** |
| --- | --- |
| Val30Met (p.Val50Met)^a^ | 2924 (49.6) |
| Wild-type | 1386 (23.5) |
| Val122Ile (p.Val142Ile) | 354 (6.0) |
| Glu89Gln (p.Glu109Gln) | 147 (2.5) |
| Thr60Ala (p.Thr80Ala) | 141 (2.4) |
| Ser50Arg (p.Ser70Arg) | 94 (1.6) |
| Ile68Leu (p.Ile88Leu) | 76 (1.3) |
| Phe64Leu (p.Phe84Leu) | 74 (1.3) |
| Ser77Tyr (p.Ser97Tyr) | 72 (1.2) |
| Ile107Val (p.Ile127Val) | 45 (0.8) |
| Asp38Ala (p.Asp58Ala) | 36 (0.6) |
| Glu89Lys (p.Glu109Lys) | 32 (0.5) |
| Gly47Ala (p.Gly67Ala) | 31 (0.5) |
| Val20Ile (p.Val40Ile) | 28 (0.5) |
| Ala97Ser (p.Ala117Ser) | 24 (0.4) |
| Leu111Met (p.Leu131Met) | 22 (0.4) |
| Val28Met (p.Val48Met) | 21 (0.4) |
| delVal122 (p.delVal142) | 19 (0.3) |
| Glu54Gln (p.Glu74Gln) | 17 (0.3) |
| His88Arg (p.His108Arg) | 17 (0.3) |
| Val122Ala (p.Val142Ala) | 16 (0.3) |
| Ser77Phe (p.Ser97Phe) | 15 (0.3) |
| Thr49Ala (p.Thr69Ala) | 15 (0.3) |
| Ser52Pro (p.Ser72Pro) | 12 (0.2) |
| Pro24Ser (p.Pro44Ser) | 11 (0.2) |
| Thr59Lys (p.Thr79Lys) | 10 (0.2) |
| Tyr114Cys (p.Tyr134Cys) | 10 (0.2) |

Genotypes recorded in ≥10 patients at enrollment shown

^a^ Includes 82 patients with Gly6Ser (p.Gly26Ser)/Val30Met (p.Val50Met) mutations
