# Supplementary material for: Clinical and genetic profile of patients enrolled in the Transthyretin Amyloidosis Outcomes Survey (THAOS): 14-year update
Source: Orphanet J Rare Dis. 2022 Jun 18;17:236. doi: 10.1186/s13023-022-02359-w (PMC9206752; doi:10.1186/s13023-022-02359-w)
Supplement: Supplementary file 2 — Additional file 2: Table 2. Distribution of phenotype in symptomatic patients according to genotype category. [file 13023_2022_2359_MOESM2_ESM.docx]

**Supplementary Table 2** Distribution of phenotype at enrollment in symptomatic patients according to genotype category

| **Phenotype category, *n* (%)** | **Overall**  **(*n* = 3779)** | **ATTRwt amyloidosis**  **(*n* = 1156)** | Val30Met overall (***n*** = 1542) | Val30Met early onset  (***n*** = 826) | Val30Met late onset  (***n*** = 588) | **Cardiac mutations**  **(*n* = 384)** | Non-Val30Met excluding cardiac  (***n*** = 697) |
| --- | --- | --- | --- | --- | --- | --- | --- |
| All symptomatic patients |  |  |  |  |  |  |  |
| Predominantly cardiac | 1539 (40.7) | 1029 (89.0) | 100 (6.5) | 27 (3.3) | 64 (10.9) | 241 (62.8) | 169 (24.2) |
| Predominantly neurologic | 1516 (40.1) | 7 (0.6) | 1123 (72.8) | 675 (81.7) | 369 (62.8) | 64 (16.7) | 322 (46.2) |
| Mixed | 628 (16.6) | 113 (9.8) | 277 (18.0) | 112 (13.6) | 142 (24.1) | 66 (17.2) | 172 (24.7) |
| No phenotype | 96 (2.5) | 7 (0.6) | 42 (2.7) | 12 (1.5) | 13 (2.2) | 13 (3.4) | 34 (4.9) |
| North America, *n* | 1136 | 668 | 29 | 1 | 25 | 256 | 183 |
| Predominantly cardiac | 830 (73.1) | 612 (91.6) | 8 (27.6) | 0 | 8 (32.0) | 167 (65.2) | 43 (23.5) |
| Predominantly neurologic | 151 (13.3) | 3 (0.4) | 14 (48.3) | 1 (100.0) | 11 (44.0) | 42 (16.4) | 92 (50.3) |
| Mixed | 131 (11.5) | 50 (7.5) | 6 (20.7) | 0 | 5 (20.0) | 39 (15.2) | 36 (19.7) |
| No phenotype | 24 (2.1) | 3 (0.4) | 1 (3.4) | 0 | 1 (4.0) | 8 (3.1) | 12 (6.6) |
| South America, *n* | 235 | 8 | 190 | 117 | 53 | 18 | 19 |
| Predominantly cardiac | 18 (7.7) | 6 (75.0) | 1 (0.5) | 0 | 1 (1.9) | 8 (44.4) | 3 (15.8) |
| Predominantly neurologic | 156 (66.4) | 1 (12.5) | 145 (76.3) | 96 (82.1) | 34 (64.2) | 3 (16.7) | 7 (36.8) |
| Mixed | 58 (24.7) | 1 (12.5) | 41 (21.6) | 20 (17.1) | 17 (32.1) | 7 (38.9) | 9 (47.4) |
| No phenotype | 3 (1.3) | 0 | 3 (1.6) | 1 (0.9) | 1 (1.9) | 0 | 0 |
| Europe, *n* | 2188 | 462 | 1212 | 668 | 477 | 108 | 406 |
| Predominantly cardiac | 640 (29.3) | 397 (85.9) | 79 (6.5) | 22 (3.3) | 51 (10.7) | 65 (60.2) | 99 (24.4) |
| Predominantly neurologic | 1106 (50.5) | 2 (0.4) | 897 (74.0) | 550 (82.3) | 309 (64.8) | 19 (17.6) | 188 (46.3) |
| Mixed | 377 (17.2) | 59 (12.8) | 200 (16.5) | 86 (12.8) | 107 (22.4) | 19 (17.6) | 99 (24.4) |
| No phenotype | 65 (3.0) | 4 (0.9) | 36 (3.0) | 10 (1.5) | 10 (2.1) | 5 (4.6) | 20 (4.9) |
| Japan, *n* | 145 | 7 | 108 | 39 | 31 | 1 | 29 |
| Predominantly cardiac | 25 (17.2) | 5 (71.4) | 12 (11.1) | 5 (12.8) | 4 (12.9) | 0 | 8 (27.6) |
| Predominantly neurologic | 80 (55.2) | 1 (14.3) | 66 (61.1) | 28 (71.8) | 14 (45.2) | 0 | 13 (44.8) |
| Mixed | 39 (26.9) | 1 (14.3) | 29 (26.9) | 6 (15.4) | 12 (38.7) | 1 (100.0) | 8 (27.6) |
| No phenotype | 1 (0.7) | 0 | 1 (0.9) | 0 | 1 (3.2) | 0 | 0 |
| Other Asia, *n* | 75 | 11 | 3 | 1 | 2 | 1 | 60 |
| Predominantly cardiac | 26 (34.7) | 9 (81.8) | 0 | 0 | 0 | 1 (100.0) | 16 (26.7) |
| Predominantly neurologic | 23 (30.7) | 0 | 1 (33.3) | 0 | 1 (50.0) | 0 | 22 (36.7) |
| Mixed | 23 (30.7) | 2 (18.2) | 1 (33.3) | 0 | 1 (50.0) | 0 | 20 (33.3) |
| No phenotype | 3 (4.0) | 0 | 1 (33.3) | 1 (100.0) | 0 | 0 | 2 (3.3) |

Val30Met early onset and late onset *n* based on all patients with available data for disease diagnosis; 128 patients with the Val30Met mutation were missing date of diagnosis. Cardiac mutations include Val122Ile, Leu111Met, Thr60Ala, and Ile68Leu. Patients with no phenotype were symptomatic patients who did fulfill criteria for any of the other phenotype categories.

ATTRwt amyloidosis = wild-type transthyretin amyloidosis
