# Supplementary material for: Clinical and genetic profile of patients enrolled in the Transthyretin Amyloidosis Outcomes Survey (THAOS): 14-year update
Source: Orphanet J Rare Dis. 2022 Jun 18;17:236. doi: 10.1186/s13023-022-02359-w (PMC9206752; doi:10.1186/s13023-022-02359-w)
Supplement: Supplementary file 3 — Additional file 3: Table 3. Clinical characteristics and patient-reported outcomes in symptomatic patients according to genotype category. [file 13023_2022_2359_MOESM3_ESM.docx]

**Supplementary Table 3** Clinical characteristics and patient-reported outcomes at enrollment in symptomatic patients according to genotype category

|  | **Overall**  **(*n* = 3779)** | **ATTRwt amyloidosis**  **(*n* = 1156)** | Val30Met early onset  (***n*** = 826) | Val30Met late onset  (***n*** = 588) | **Cardiac mutations**  **(*n* = 384)** | Non-Val30Met excluding cardiac  (***n*** = 697) |
| --- | --- | --- | --- | --- | --- | --- |
| BMI (kg/m^2^), *n* | 3658 | 1125 | 820 | 577 | 365 | 675 |
| Mean (SD) | 25.8 (13.7) | 28.1 (19.1) | 22.9 (4.7) | 25.5 (12.3) | 27.6 (17.9) | 24.9 (7.3) |
| Modified BMI^a^ (g/L), *n* | 2462 | 715 | 654 | 436 | 167 | 426 |
| Mean (SD) | 1027.5 (230.5) | 1072.2 (204.7) | 992.4 (235.7) | 1013.6 (226.4) | 1057.3 (227.3) | 995.5 (244.5) |
| Sitting systolic BP, *n* | 3636 | 1118 | 814 | 572 | 360 | 672 |
| Mean (SD) | 121.9 (18.8) | 120.3 (17.2) | 123.0 (15.1) | 132.8 (20.3) | 116.5 (18.0) | 116.7 (19.9) |
| Sitting diastolic BP, *n* | 3634 | 1118 | 814 | 571 | 359 | 672 |
| Mean (SD) | 75.1 (12.0) | 72.6 (11.6) | 78.5 (11.3) | 78.2 (11.3) | 72.5 (11.2) | 73.8 (12.8) |
| Derived NIS-LL score, *n* | 1505 | 143 | 616 | 277 | 74 | 335 |
| Mean (SD) | 17.9 (21.2) | 4.3 (6.2) | 17.6 (20.0) | 29.8 (24.0) | 7.1 (9.2) | 17.7 (21.2) |
| Reflex score, *n* | 2359 | 298 | 773 | 469 | 170 | 560 |
| Mean (SD) | 7.7 (3.3) | 9.3 (1.8) | 8.5 (2.4) | 6.1 (3.7) | 8.6 (2.5) | 6.8 (3.8) |
| Motor score, *n* | 2243 | 393 | 736 | 385 | 157 | 489 |
| Mean (SD) | 151.0 (20.0) | 158.5 (11.9) | 151.2 (18.9) | 143.9 (23.9) | 155.8 (17.7) | 149.2 (20.6) |
| Sensory score, *n* | 1295 | 103 | 548 | 245 | 58 | 287 |
| Mean (SD) | 101.6 (26.7) | 121.8 (6.5) | 98.6 (25.4) | 87.3 (31.1) | 117.1 (13.0) | 108.6 (21.5) |
| LV septum thickness (mm), *n* | 1993 | 880 | 182 | 237 | 215 | 432 |
| Mean (SD) | 15.9 (5.3) | 17.4 (3.5) | 9.9 (1.8) | 15.1(4.3) | 16.8 (4.1) | 16.0 (7.8) |
| LV ejection fraction (%), *n* | 1869 | 885 | 153 | 160 | 230 | 409 |
| Mean (SD) | 52.2 (13.9) | 48.1 (12.5) | 63.3 (10.0) | 60.5 (10.0) | 46.3 (15.2) | 56.0 (13.6) |
| EQ-5D-3L: VAS overall health, *n* | 2423 | 743 | 645 | 376 | 206 | 390 |
| Mean (SD) | 65.2 (20.5) | 65.5 (19.3) | 69.4 (18.5) | 59.7 (22.0) | 65.0 (22.2) | 62.6 (22.0) |
| EQ-5D-3L: Derived index, *n* | 2461 | 753 | 651 | 378 | 214 | 400 |
| Mean (SD) | 0.7 (0.2) | 0.8 (0.2) | 0.8 (0.2) | 0.7 (0.2) | 0.8 (0.2) | 0.7 (0.2) |
| Norfolk Total QoL | 2472 | 742 | 656 | 390 | 210 | 411 |
| Mean (SD) | 33.3 (29.2) | 23.9 (20.9) | 30.7 (26.6) | 48.8 (33.5) | 29.2 (27.4) | 42.2 (33.9) |
| Karnofsky Performance Status, *n* (%) |  |  |  |  |  |  |
| 10–30 | 10 (0.2) | 2 (0.2) | 0 | 0 | 3 (0.8) | 4 (0.6) |
| 40–60 | 429 (11.4) | 82 (7.1) | 71 (8.6) | 125 (21.3) | 40 (10.4) | 101 (14.5) |
| 70–90 | 1904 (50.4) | 375 (32.4) | 642 (77.7) | 346 (58.8) | 149 (38.8) | 355 (50.9) |
| 100 | 212 (5.6) | 31 (2.7) | 39 (4.7) | 23 (3.9) | 13 (3.4) | 81 (11.6) |

Val30Met early onset and late onset *n* based on all patients with available data for disease diagnosis; 128 patients with the Val30Met mutation were missing date of diagnosis. Cardiac mutations included Val122Ile, Leu111Met, Thr60Ala, and Ile68Leu

^a^ Calculated by multiplying BMI by serum albumin levels to compensate for fluid accumulation

ATTR amyloidosis = transthyretin amyloidosis; ATTRwt amyloidosis = wild-type transthyretin amyloidosis; BMI = body mass index; BP = blood pressure; LV = left ventricular; mPND = modified Polyneuropathy Disability; NIS-LL = Neuropathy Impairment Score in the Lower Limbs; QoL = quality of life; VAS = visual analog scale
