# Supplementary material for: Clinical and genetic profile of patients enrolled in the Transthyretin Amyloidosis Outcomes Survey (THAOS): 14-year update
Source: Orphanet J Rare Dis. 2022 Jun 18;17:236. doi: 10.1186/s13023-022-02359-w (PMC9206752; doi:10.1186/s13023-022-02359-w)
Supplement: Supplementary file 4 — Additional file 4: Table 4. Neurologic findings in symptomatic patients with a predominantly neurologic or mixed phenotype. [file 13023_2022_2359_MOESM4_ESM.docx]

**Supplementary Table 4** Neurologic characteristics at enrollment in symptomatic patients with a predominantly neurologic or mixed phenotype

|  | **Overall**  **(*n* = 2144)** | **ATTRwt amyloidosis**  **(*n* = 120)** | Val30Met early onset  (***n*** = 787) | Val30Met late onset  (***n*** = 511) | **Cardiac mutations**  **(*n* = 130)** | Non-Val30Met excluding cardiac  (***n*** = 494) |
| --- | --- | --- | --- | --- | --- | --- |
| Patients with data available, *n* | 1755 | 50 | 739 | 437 | 81 | 359 |
| mPND score at enrollment, *n* (%) |  |  |  |  |  |  |
| 0 | 165 (9.4) | 20 (40.0) | 46 (6.2) | 11 (2.5) | 18 (22.2) | 57 (15.9) |
| I | 914 (52.1) | 13 (26.0) | 485 (65.6) | 184 (42.1) | 37 (45.7) | 148 (41.2) |
| II | 364 (20.7) | 10 (20.0) | 149 (20.2) | 118 (27.0) | 6 (7.4) | 69 (19.2) |
| IIIa | 138 (7.9) | 4 (8.0) | 33 (4.5) | 56 (12.8) | 10 (12.3) | 27 (7.5) |
| IIIb | 106 (6.0) | 2 (4.0) | 15 (2.0) | 39 (8.9) | 8 (9.9) | 37 (10.3) |
| IV | 68 (3.9) | 1 (2.0) | 11 (1.5) | 29 (6.6) | 2 (2.5) | 21 (5.8) |

Val30Met early onset and late onset *n* based on all patients with available data for disease diagnosis; 128 patients with the Val30Met mutation were missing date of diagnosis.

ATTRwt amyloidosis = wild-type transthyretin amyloidosis; mPND = modified Polyneuropathy Disability
